# Supplementary material for: Analysis of EVs from patients with advanced pancreatic cancer identifies antigens and miRNAs with predictive value
Source: Mol Ther Methods Clin Dev. 2023 May 11;29:473–82. doi: 10.1016/j.omtm.2023.05.009 (PMC10238807; doi:10.1016/j.omtm.2023.05.009)
Supplement: Document S1. Figures S1–S4 and Table S1 [file mmc1.pdf]

OMTM, Volume *29*

Supplemental information

Analysis of EVs from patients with advanced pancreatic cancer identifies antigens and miRNAs with predictive value

Ivan Vannini, Tania Rossi, Mattia Melloni, Martina Valgiusti, Milena Urbini, Alessandro Passardi, Giulia Bartolini, Chiara Gallio, Irene Azzali, Sara Bandini, Valentina Ancarani, Lorenzo Montanaro, Giovanni Luca Frassineti, Francesco Fabbri, and Ilario Giovanni Rapposelli

Supplemental Material

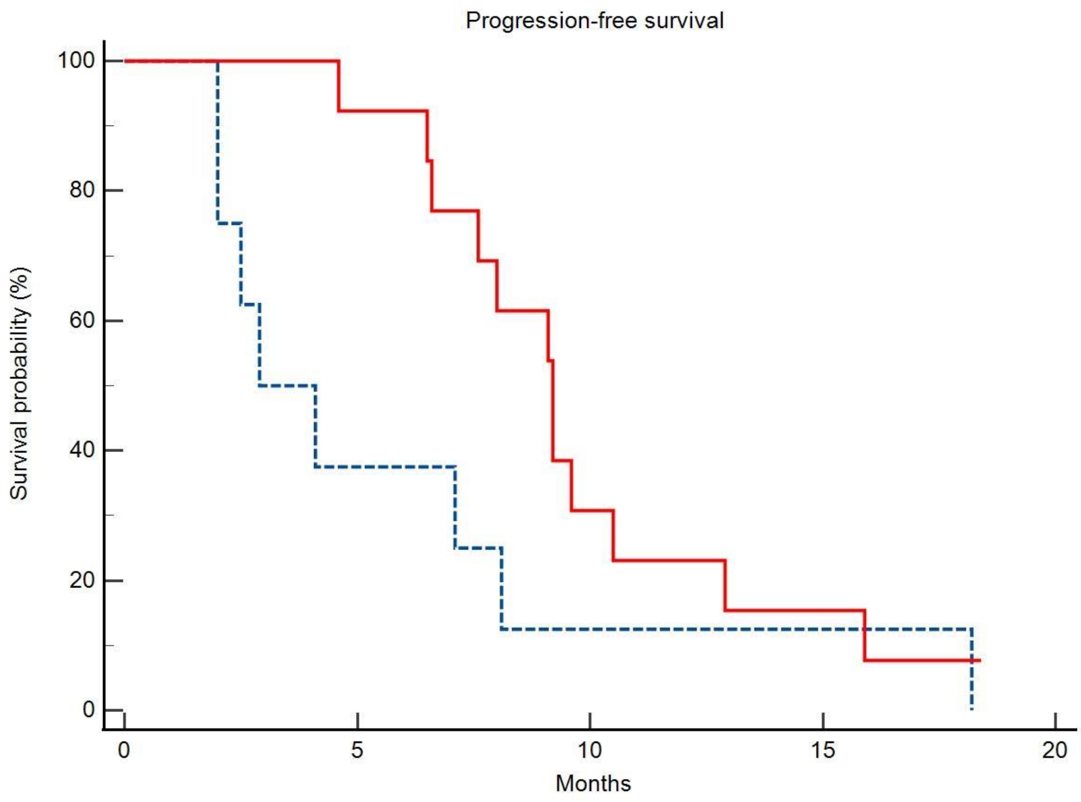

Figure S1. Kaplan-Meier analysis of progression-free survival. Median progression-free survival was 9.2 months for responders and 2.9 months for non-responders (hazard ratio 0.39, 95% confidence interval 0.13-1.12; p = 0.08). Red line: responders; blue line: non-responders.

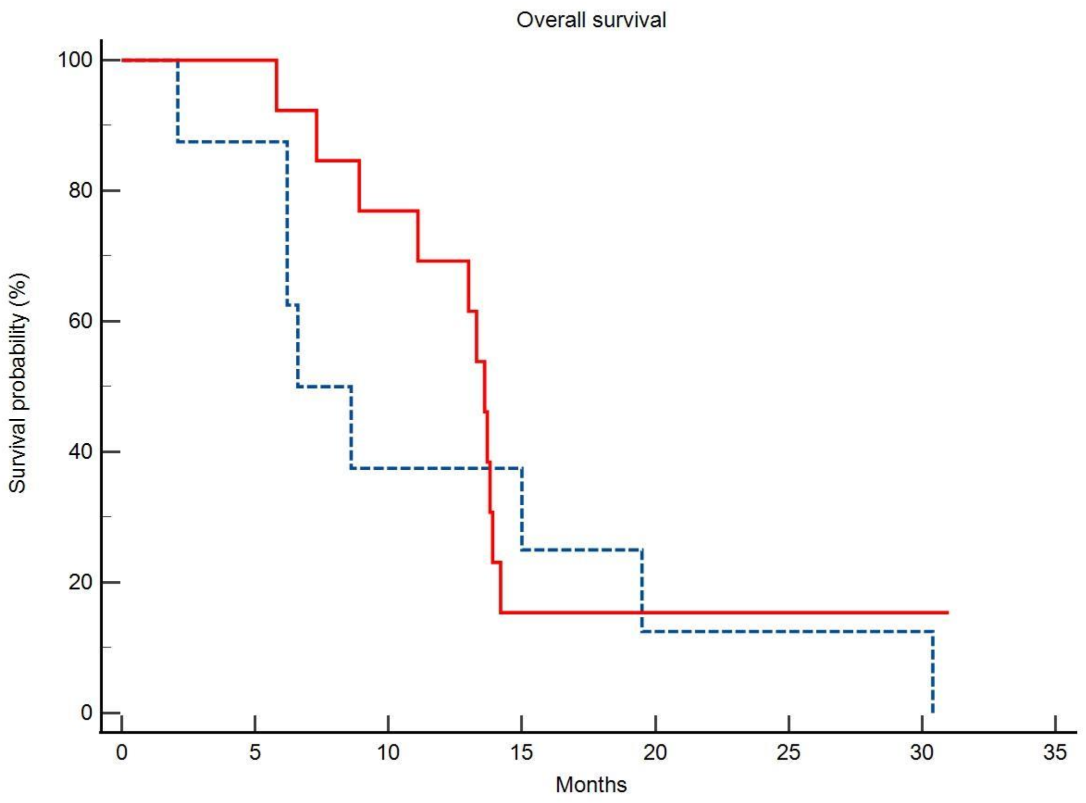

Figure S2. Kaplan-Meier analysis of overall survival. Median overall survival was 13.6 months for responders and 6.6 months for non-responders (hazard ratio 0.77, 95% confidence interval 0.29-2.04; p = 0.60). Red line: responders; blue line: non-responders.

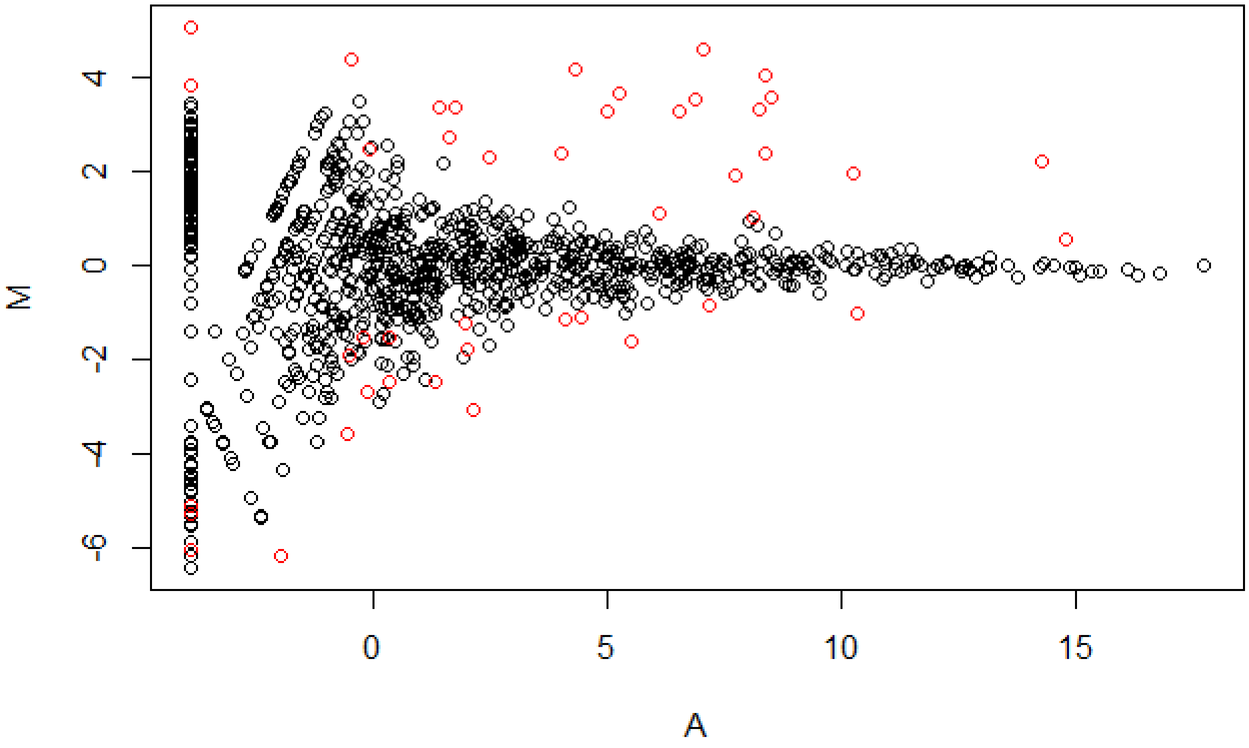

Figure S3. MA-plot displaying the log fold change (M) against the log abundance (A) of microRNAs (miRNAs). Red dots identify the differentially expressed miRNAs (p-value < 0.05) between responder and non-responder advanced pancreatic cancer patients, as described in the main text.

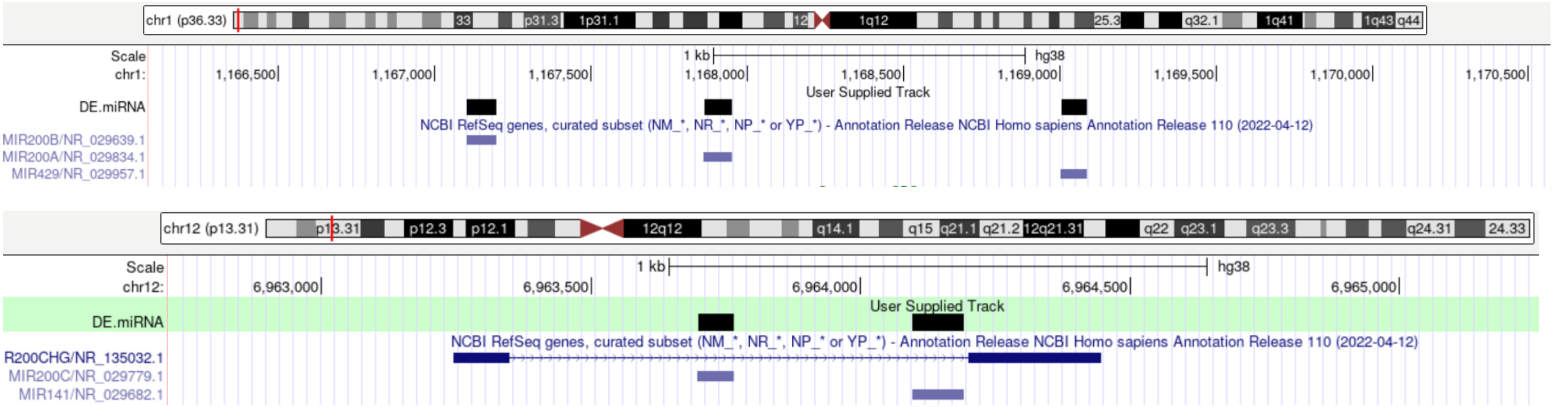

Figure S4. Genomic localization of miR-200 family members in chromosome 1 (miR-200a, miR-200b, miR-429) and chromosome 12 (miR-200c, miR-141). “DE.miRNA”= UCSC tracks of differentially expressed miRNAs found in responder vs non-responser PC patients.

Table S1. List of differentially expressed microRNAs (miRNAs) between responder and non-responder pancreatic cancer patients.

miRNA	Fold change	Log fold change	*P*-value	Genomic position (GRCh38) Chromosome: start-end [strand]
hsa-miR-375-3p	23,98	4,58	2,685E-08	chr2: 219001645-219001708 [-]
hsa-miR-200a-5p	17,63	4,14	1,682E-02	chr1: 1167863-1167952 [+]
hsa-miR-135b-5p	17,47	4,13	1,029E-05	chr1: 205448302-205448398 [-]
hsa-miR-200c-3p	16,07	4,01	1,593E-08	chr12: 6963699-6963766 [+]
hsa-miR-429	12,22	3,61	1,728E-06	chr1: 1169005-1169087 [+]
hsa-miR-141-3p	11,92	3,58	3,298E-07	chr12: 6964097-6964191 [+]
hsa-miR-196a-5p	11,40	3,51	4,546E-05	chr17: 48632490-48632559 [-]
hsa-miR-545-5p	10,40	3,38	3,949E-02	chrX: 74287104-74287209 [-]
hsa-miR-215-5p	9,79	3,29	8,493E-06	chr1: 220117853-220117962 [-]
hsa-miR-200a-3p	9,76	3,29	2,736E-06	chr1: 1167863-1167952 [+]
hsa-miR-200b-3p	9,46	3,24	2,220E-06	chr1: 1167104-1167198 [+]
hsa-miR-141-5p	6,96	2,80	1,443E-02	chr12: 6964097-6964191 [+]
hsa-miR-885-3p	5,90	2,56	8,151E-03	chr3: 10394489-10394562 [-]
hsa-miR-1268a	5,47	2,45	4,396E-02	chr15: 22225278-22225329 [-]
hsa-miR-194-5p	5,15	2,37	6,794E-06	chr1: 220118157-220118241 [-]
hsa-miR-200b-5p	4,85	2,28	2,589E-02	chr1: 1167104-1167198 [+]
hsa-miR-122-3p	4,66	2,22	2,479E-03	chr18: 58451074-58451158 [+]
hsa-miR-122-5p	4,56	2,19	4,346E-05	chr18: 58451074-58451158 [+]
hsa-miR-589-3p	4,01	2,00	3,304E-02	chr7: 5495819-5495917 [-]
hsa-miR-192-5p	3,88	1,96	5,158E-05	chr11: 64891137-64891246 [-]
hsa-miR-455-5p	3,79	1,92	1,972E-02	chr9: 114209434-114209529 [+]
hsa-miR-92b-3p	3,75	1,91	1,306E-03	chr1: 155195177-155195272 [+]
hsa-miR-100-5p	2,13	1,09	3,032E-02	chr11: 122152229-122152308 [-]
hsa-miR-483-5p	2,00	1,00	2,277E-02	chr11: 2134134-2134209 [-]
hsa-miR-21-5p	1,46	0,55	4,339E-02	chr17: 59841266-59841337 [+]
hsa-miR-411-5p	-1,80	-0,85	2,981E-02	chr14: 101023325-101023420 [+]
hsa-miR-125b-5p	-2,02	-1,01	2,062E-02	chr11: 122099757-122099844 [-]
hsa-miR-136-5p	-2,06	-1,04	2,383E-02	chr14: 100884702-100884783 [+]
hsa-miR-1249-3p	-2,14	-1,10	3,642E-02	chr22: 45200954-45201019 [-]
hsa-miR-19b-1-5p	-2,39	-1,26	3,636E-02	chr13: 91351192-91351278 [+]
hsa-miR-1185-1-3p	-2,49	-1,32	1,769E-02	chr14: 101042977-101043062 [+]
hsa-miR-190b-5p	-3,07	-1,62	7,568E-03	chr1: 154193665-154193743 [-]
hsa-miR-496	-3,32	-1,73	1,291E-02	chr14: 101060573-101060674 [+]
hsa-miR-18b-5p	-3,56	-1,83	3,640E-02	chrX: 134170041-134170111 [-]
hsa-miR-12135	-3,56	-1,83	2,872E-02	chr15: 73299910-73299979 [+]
hsa-miR-6131	-3,93	-1,97	3,826E-02	chr5: 10478037-10478145 [+]
hsa-miR-516a-5p	-3,93	-1,97	3,690E-02	chr19: 53756741-53756830 [+]
hsa-miR-3161	-4,55	-2,19	3,920E-02	chr11: 48096782-48096858 [+]
hsa-miR-6806-3p	-5,86	-2,55	4,309E-02	chr19: 58334688-58334751 [+]
hsa-miR-4451	-5,86	-2,55	4,307E-02	chr4: 85722468-85722533 [+]
hsa-miR-411-3p	-5,90	-2,56	4,681E-02	chr14: 101023325-101023420 [+]
hsa-miR-216a-5p	-6,36	-2,67	3,178E-02	chr2: 55988950-55989059 [-]
hsa-miR-362-3p	-6,37	-2,67	2,990E-02	chrX: 50008964-50009028 [+]
hsa-miR-146a-3p	-7,28	-2,86	2,022E-04	chr5: 160485352-160485450 [+]
