# Supplementary material for: Analysis of EVs from patients with advanced pancreatic cancer identifies antigens and miRNAs with predictive value
Source: Mol Ther Methods Clin Dev. 2023 May 11;29:473–82. doi: 10.1016/j.omtm.2023.05.009 (PMC10238807; doi:10.1016/j.omtm.2023.05.009)
Supplement: Document S2. Article plus supplemental information [file mmc2.pdf]

Molecular Therapy
Methods & Clinical Development
Original Article

Analysis of EVs from patients with advanced pancreatic cancer identifies antigens and miRNAs with predictive value

Ivan Vannini,[1] Tania Rossi,[1] Mattia Melloni,[2] Martina Valgiusti,[3] Milena Urbini,[1] Alessandro Passardi,[3] Giulia Bartolini,[3] Chiara Gallio,[3] Irene Azzali,[4] Sara Bandini,[1] Valentina Ancarani,[5] Lorenzo Montanaro,[6,7] Giovanni Luca Frassineti,[3] Francesco Fabbri,[1] and Ilario Giovanni Rapposelli[3]

[1]Biosciences Laboratory, IRCCS Istituto Romagnolo per lo Studio dei Tumori (IRST) "Dino Amadori", Meldola, Italy; [2]Department of Translational Medicine, University of Ferrara, Ferrara, Italy; [3]Department of Medical Oncology, IRCCS Istituto Romagnolo per lo Studio dei Tumori (IRST) "Dino Amadori", Meldola, Italy; [4]Unit of Biostatistics and Clinical Trials, IRCCS Istituto Romagnolo per lo Studio dei Tumori (IRST) "Dino Amadori", Meldola, Italy; [5]Immunotherapy-Cell Therapy and Biobank Unit, IRCCS Istituto Romagnolo per lo Studio dei Tumori (IRST) "Dino Amadori", Meldola, Italy; [6]Department of Medical and Surgical Sciences (DIMEC), Alma Mater Studiorum - University of Bologna, Bologna, Italy; [7]Departmental Program in Laboratory Medicine, IRCCS Azienda Ospedaliero-Universitaria di Bologna, Bologna, Italy

The identification of predictive factors for treatment of pancreatic cancer (PC) is an unmet clinical need. In the present work, we analyzed blood-derived extracellular vesicles (EVs) from patients with advanced PC in order to find a molecular signature predictive of response to therapy. We analyzed samples from 21 patients with advanced PC, all receiving first-line treatment with gemcitabine + nab-paclitaxel. Isolated EVs have been analyzed, and the results of laboratory have been matched with clinical data in order to investigate possible predictive factors. EV concentration and size were similar between responder and non-responder patients. Analysis of 37 EV surface epitopes showed a decreased expression of SSEA4 and CD81 in responder patients. We detected more than 450 expressed miRNAs in EVs. A comparative survey between responder and non-responder patients showed that at least 44 miRNAs were differently expressed. Some of these miRNAs have already been observed in relation to the survival and gemcitabine sensitivity of tumor cells. In conclusion, we showed the ability of our approach to identify EV-derived biomarkers with predictive value for therapy response in PC. Our findings are worthy of further investigation, including the analysis of samples from patients treated with different schedules and in different settings.

INTRODUCTION

Pancreatic cancer (PC) is the seventh leading cause of cancer death worldwide,[1] with a 5-year survival of about 11%.[2] Indeed, the majority of patients receive diagnosis at advanced stage, only amenable to systemic treatment.[3] Recent advancements brought combination chemotherapy regimens such as FOLFIRINOX, gemcitabine + nab-paclitaxel, and PAXG, which are current options in first-line treatment of metastatic and locally advanced disease, and they have improved survival in this setting.[4–6] Nevertheless, response rates with the above-mentioned regimes range from 23% to 50%.[5,6] Furthermore, disease progression often comes along with a deterioration in patient's performance status, and only about 49% of patients receive second-line treatment.[7] Thus, response to first-line treatment is crucial for the whole patient journey; consequently, the availability of factors able to predict a single patient's sensitivity to different chemotherapy regimens would be crucial. Currently, no predictive factors exist to guide treatment selection in advanced PC, with the exception of DNA damage repair alterations such as mutations of *BRCA1*, *BRCA2*, and *PALB2*. Indeed, *BRCA1/2* and *PALB2* mutations confer sensitivity to platinum-containing chemotherapy,[8,9] and germline *BRCA1/2* mutations open the possibility to maintenance treatment with olaparib.[10]

Nowadays, several studies try to exploit a liquid biopsy approach in order to investigate circulating factors with predictive value in PC.[11] Extracellular vesicles (EVs) are small particles released by cells that can be easily isolated from blood. EVs contain biomolecules such as DNA, RNA, and proteins and facilitate intercellular communication. They are able to transfer their content into target cells and regulate several cellular functions, e.g., proliferation, apoptosis, and migration.[12] EVs are secreted at a higher amount by cancer cells,[13] and they have a key role in cancer biology. Indeed, they are involved in the regulation of several hallmarks of cancer: specifically in PC, they have been involved in cell proliferation, promotion of invasion and metastases, modulation of tumor-associated immunity, and chemoresistance.[14]

Received 19 October 2022; accepted 9 May 2023;
https://doi.org/10.1016/j.omtm.2023.05.009.

Correspondence: Tania Rossi, Biosciences Laboratory, IRCCS Istituto Romagnolo per lo Studio dei Tumori (IRST) "Dino Amadori", Via P. Maroncelli 40, Meldola, FC 47014, Italy.
E-mail: tania.rossi@irst.emr.it

© 2023 The Author(s).
This is an open access article under the CC BY license (http://creativecommons.org/licenses/by/4.0/).

In EV-mediated chemoresistance, a key role is played by their content in microRNAs (miRNAs), non-protein-coding RNA fragments that regulate the expression of target proteins through degradation of messenger RNA or interference with the translational process.[15] EVs from cancer cells are enriched in miRNAs,[16] which are involved in chemoresistance of tumor cells by targeting drug-resistance-related genes or influencing genes related to cell proliferation, cell cycle, and apoptosis.[17] For example, PC cells incubated with gemcitabine upregulate miR-155, which is transferred to other PC cells via EVs and is able to promote gemcitabine resistance through facilitation of anti-apoptotic activity and suppression of deoxycytidine kinase, a key gemcitabine-metabolizing enzyme;[18] EVs released by cancer-associated fibroblasts contribute to gemcitabine resistance through the upregulation of chemoresistance-inducing factor Snail and its target miR-146a in recipient PC cells.[19] Recently, we have analyzed the miRNA content of different plasma circulating fractions demonstrating the feasibility of EV-derived miRNA profiling.[20]

Given the role of EV-derived antigens and miRNAs in chemoresistance of tumor cells, here we propose to analyze the miRNA content but also the surface antigens of EVs from patients with advanced PC, collected before first-line treatment with gemcitabine + nab-paclitaxel, with the aim to investigate a molecular signature able to predict response to treatment.

Table 1. Patient characteristics

	n	%
Age (years)	–	–
Median 70 (range 54–82)	–	–
Sex	–	–
Female	13	62
Male	8	38
Stage	–	–
Metastatic	12	57
Locally advanced	9	43
Best response	–	–
Complete response	0	–
Partial response	13	62
Stable disease	4	19
Progressive disease	4	19
Baseline CA19.9	–	–
≤[a]ULN	5	24
>[a]ULN and <5 [a]ULN	1	5
≥5 [a]ULN	15	71

[a]ULN, upper limit of normal.

RESULTS

Patient characteristics

Patient characteristics are summarized in Table 1. Of 21 patients analyzed, 12 had metastatic disease, and nine had locally advanced disease. As for best response assessment during treatment with gemcitabine + nab-paclitaxel, 13 patients reported an objective response (partial response, none had a complete response), whereas eight were classified as non-responders (four with stable disease, four with progressive disease). Median progression-free survival was 9.2 months for responders and 2.9 months for non-responders (hazard ratio 0.39, 95% confidence interval 0.13–1.12; $p = 0.08$) (Figure S1). Median overall survival was 13.6 months for responders and 6.6 months for non-responders (hazard ratio 0.77, 95% confidence interval 0.29–2.04; $p = 0.60$) (Figure S2).

Nanoparticle tracking analysis of EVs

EVs have been isolated by plasma of PC patients through size exclusion chromatography (SEC). Then, nanoparticle tracking analysis (NTA) has been performed with NanoSight instrument for EV concentration and size. A representative NTA for each group is shown in Figure 1A.

We analyzed the relative concentration of EVs with a diameter between 50 and 150 nm in the two groups of patients, noting no significant difference (responders median 0.68, range 0.52–0.86 vs. non-responders median 0.73, range 0.52–0.82; $p = 0.59$) (Figure 1B). Also, the relative concentration of EVs with a diameter between 150 and 300 nm did not show any significant difference between the two groups (responders median 0.30, range 0.13–0.45 vs. non-responders median 0.26, range 0.18–0.45; $p = 0.5$) (Figure 1C). The median of mean EV diameter in the responder patients was 134.3 nm, range 108.2–150.4 nm, and the median of mode diameter was 108.5 nm, range 78.5–126.5 nm. In the non-responder group the median of mean diameter was 132.5 nm, range 119.3–147.1 nm, and the median of mode diameter 105 nm, range 81.5–134.5 nm. We did not observe any significant difference between the median of mean EV diameters ($p = 0.5$) and the median of EV mode diameters ($p = 0.98$) between the two patient groups (Figures 1D and 1E).

Characterization of surface EV markers

Then, surface protein expression analysis was performed on the EVs using a protein multiplex bead-based flow cytometry assay as previously reported.[21] The median fluorescence intensity (MFI) of each surface marker is indicated in Figure 2A. Expression of typical exosomal markers (CD9, CD63, CD81) was observed in both patient groups.

Among the analyzed EV surface proteins, we observed that the MFIs of CD81 and SSEA4 were higher in non-responders than in responders ($p < 0.03$) (Figure 2B). Other markers on the EV surface were differentially expressed between the two patient groups such as CD42a, CD41b, CD29, CD62P, CD40, CD9, CD31, CD63, HLA-DR, CD69, HLA-BC, CD49e, CD105, CD133/1, CD25, CD11c, and CD14, but the differences were not statistically significant (Figure 2A). CD3 was completely absent from the vesicles of the PC patient.

MicroRNA cargo of patient EVs

Finally, we analyzed the miRNA content in EVs isolated from PC patients. To perform this analysis, we extracted the RNA from EVs, and then we produced the small libraries that were sequenced in NextSeq550 instrument (Illumina).

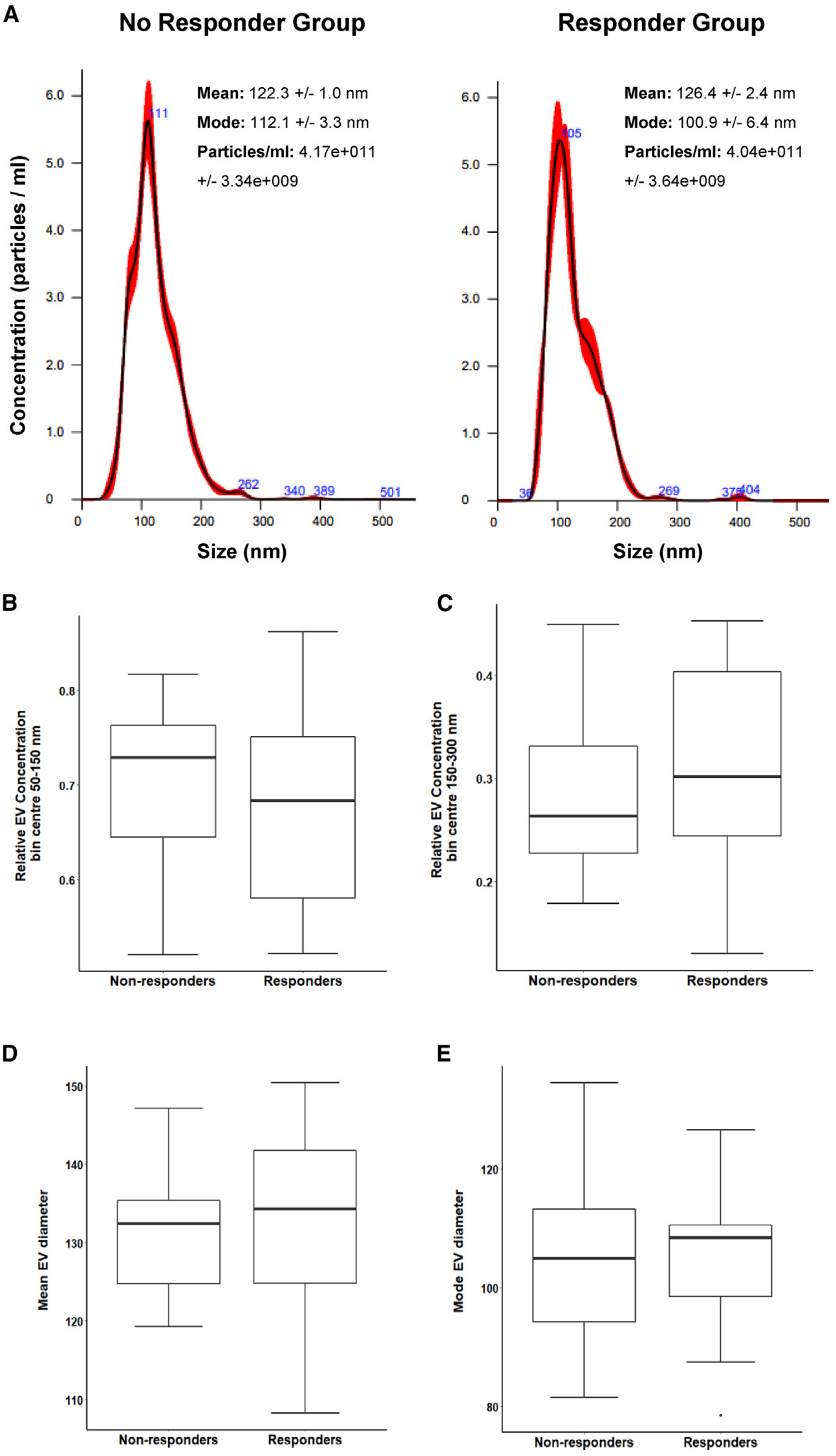

Figure 1. Nanoparticle tracking analysis (NTA) of extracellular vesicles (EVs)

(A) NTA profile analysis of EV-enriched SEC fractions obtained from plasma of responder and non-responder PC patients. A representative graph is reported for each group. (B) Relative concentrations of EVs with 50–150 nm diameter (50–150 nm EV concentrations/total EV concentrations) in responder and non-responder PC patients ($p = 0.59$). (C) Relative concentrations of EVs with 150–300 nm diameter (150–300 nm EV concentrations/total EV concentrations) in responder and non-responder PC patients ($p = 0.5$). (D) Mean EV diameter in responder and non-responder PC patients ($p = 0,5$). (E) Mode EV diameter in responder and non-responder PC patients ($p = 0.98$).

Through this analysis, 44 miRNAs (Table S1) were differentially expressed between responder and non-responder patients, and these differences were significant ($p < 0.05$). Of these miRNAs, 25 were upregulated in responder patients, and the most expressed miRNAs had a $\log_2$ fold change between 4.58 and 3.24. The remaining 19 miRNAs were downregulated in responders, and the least expressed miRNAs had a $\log_2$ fold change ranging from −2.86 to −1.83 (Figures 3 and S3).

We searched the literature for the role in PC of the more dysregulated miRNAs that we identified in responder versus non-responder patients (Figure 4), and the function of the identified miRNAs is summarized in Table 2. Interestingly all the members of the miR-200 family (miR-200a, miR-200b, miR-200c, miR-141, and miR-429), which are localized in two clusters in chromosomes 1 and 12, emerged as upregulated in responders (Figure S4).

To confirm the data obtained with NGS, we analyzed through real-time PCR the expression of two selected miRNAs (miR-200c-3p and miR-375-3p) starting from the RNA samples used for library preparation. As shown in Figure 5, we observed a statistically significant upregulation of miR-200c-3p ($p = 0.0199$) and miR-375-3p ($p = 0.0446$) in EVs from responder patients compared with non-responder patients, thus confirming NGS data.

DISCUSSION

PC is characterized by a poor prognosis. Despite the recent advancements deriving from combination regimens currently available, the 5-year survival of metastatic disease is about 3% ([2]). Furthermore, even with the most active regimens, response rates are 23% (gemcitabine + nab-paclitaxel), 31.6% (FOLFIRINOX), and 50% (PAXG).[4–6] These figures highlight the existence of a remarkable amount of therapy resistance, intrinsic or acquired. Given these premises and taking

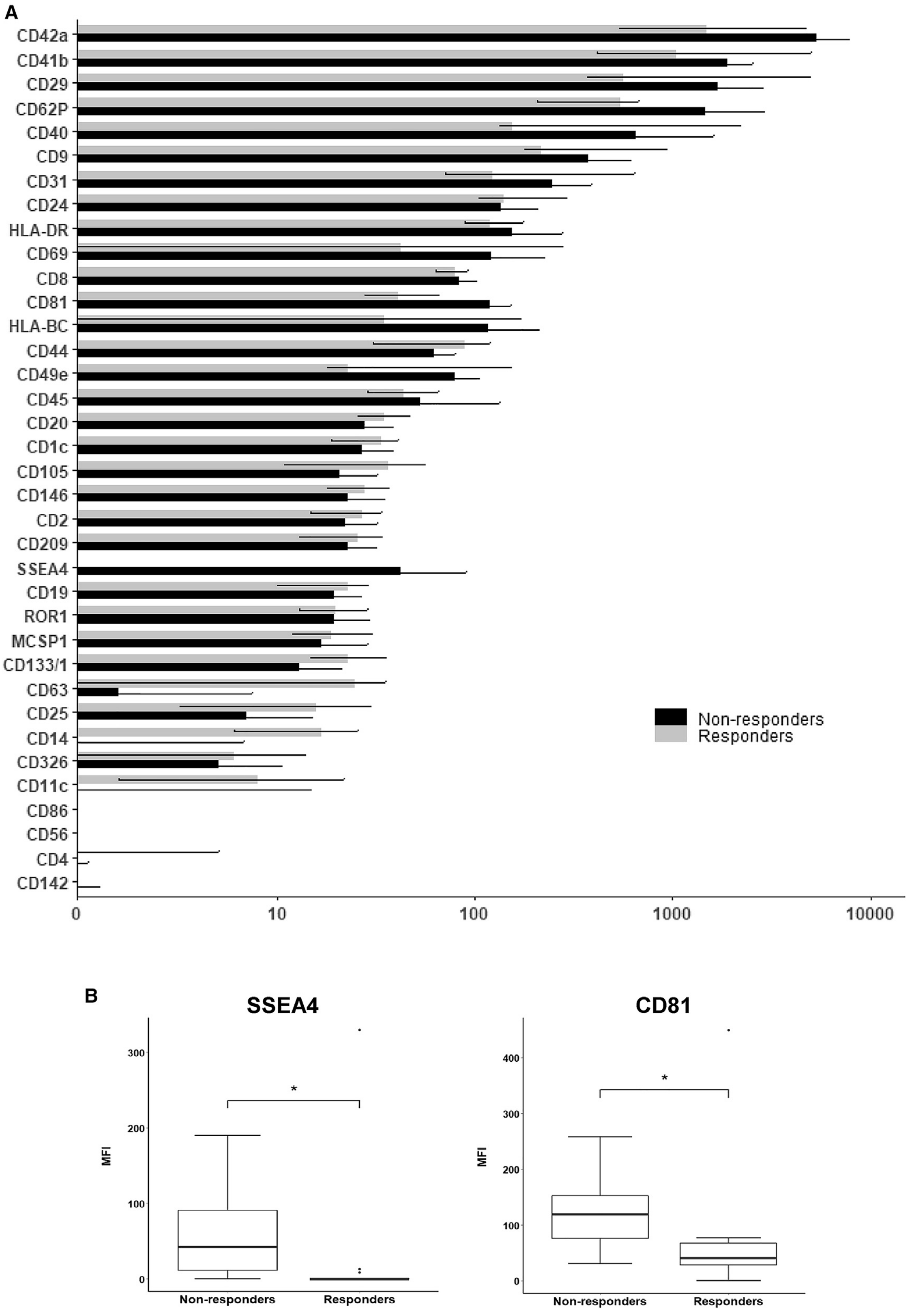

(legend on next page)

into account the low number of patients that receive second-line therapy due to a deterioration in clinical status upon disease progression,[7] the identification of predictive markers is crucial to identify patients with more chances to benefit or not from first-line treatment. This is an unmet clinical need, since the identification of such markers would allow a better selection of first-line therapy for each patient, in a personalized medicine approach aimed to improve outcomes and reduce unnecessary side effects.

Here we show the feasibility of a liquid biopsy approach to create an EV profile that can help to identify patients who respond to gemcitabine + nab-paclitaxel therapy. We isolated EVs in plasma collected before therapy and compared findings of laboratory analyses in responder and non-responder patients, identified according to RECIST 1.1.[31] NTA did not show any significant difference between the two groups of patients in terms of EV size and number (Figures 1A–1E), probably due to the low size of PC samples. We then proceeded with the analysis of EV surface antigens, using a multiplexed phenotyping cytofluorimetric approach able to observe 37 antigens (Figure 2A). We observed that the SSEA4 was less expressed in responder than in non-responder patients (Figure 2B). SSEA4 is a cell-surface glycosphingolipid used to distinguish human embryonic stem cells and human embryonal carcinoma cells or induced pluripotent stem cells.[32,33] In the literature, it has been observed that high levels of SSEA4 in tissue of PC patients are correlated with a poor survival rate as observed by Lin et al.[34] To confirm the oncogenic capacity of SSEA4, these authors constructed chimeric anti-SSEA4 monoclonal antibodies that are highly effective against PC *in vitro* and *in vivo*. These data were confirmed by the construction of anti-SSEA4 CAR-T cells that are able to eliminate PC cells in cell and animal studies.

The presence of SSEA4 in EV surfaces may indicate a derivation of the EVs from PC cells highly resistant to therapy and able to convey pro-tumor information in the microenvironment determining a poor chemotherapy response in PC patients. CD81 also is more expressed in the EVs of the non-responders than in the responder patients (Figure 2B). This protein belongs to the tetraspanin family characterized by four hydrophobic domains. It is an important surface protein in signal transduction. In a recent study by Quagliano et al., it has been shown that CD81 knockout causes increased chemosensitivity in pediatric hematological malignancies.[35] This chemosensitization is mediated by the control of Bruton tyrosine kinase signaling and the induction of p53-mediated cell death in leukemic cells. CD81 may be a predictive marker of response to chemotherapy in PC patients thought the analysis of its EV expression level. Further studies on the mechanism that determines CD81-induced chemoresistance will be needed to confirm this hypothesis.

The RNA-seq analysis of the miRNA content of EVs showed a different expression of miRNAs in the two groups of patients (responders and non-responders): we observed that 44 miRNAs were dysregulated, with a probable oncogene or tumor suppressor function (Figure 3). Interestingly, we found through our analysis that the members of the miR-200 family (miR-200a, miR-200b, miR-200c, miR-141, and miR-429) were significantly upregulated in EVs from responder compared with non-responder PC patients. This family is widely studied in a plethora of tumor types, and besides epithelial-mesenchymal transition (EMT), it is now recognized as a key player in different signaling pathways governing apoptosis, drug resistance, cell proliferation, migration, and invasiveness.[36] Among the members of the miR-200 family described in PC, miR-200c-3p was demonstrated in the literature as a tumor suppressor, since increased expression in PC specimens was associated with better survival rate.[23] In our study, we also observed an upregulation of miR-200b-3p in the EVs of responder patients (Figure 4). High miR-200b-3p expression in PC cells resulted in increased chemosensitivity to gemcitabine and induced a mesenchymal to epithelial transition inhibiting *ZEB1* gene as shown in the literature.[25,26] Li et al. observed that gemcitabine-resistant PC cells had low expression of miR-200c-3p and miR-200b-3p, and re-expression of both miRNAs through transfection studies led to an inhibition of genes encoding for ZEB1, slug, and vimentin, which are involved in the processes of EMT that is associated with drug resistance.[24] The low expression of these miRNAs in the EVs of non-responder patients may explain their worse outcome in our study. The low expression of these miRNAs inside of EVs of the tumor microenvironment could activate pro-tumor factors and chemoresistance processes in host cells. We found other miRNAs upregulated in the EVs of the responders, and these miRNAs have a tumor suppressor function as confirmed by several research groups. Overexpression of miR-200a-3p caused an inhibition of growth and invasiveness. This effect was determined by miR-200a-3p targeting on β-catenin and consequent inhibition of its signaling.[30] Overexpression of miR-429 inhibited cell proliferation in PC by targeting the TANK binding kinase 1, a protein that acts as an activator of the oncogenic Akt kinase and of the KRAS pathway.[27] miR-141-3p and miR-141-5p exercise their tumor suppressor function by targeting transmembrane-4-L-six-family-1 (TM4SF1), a small 22-kDa four-transmembrane-domain protein, determining an inhibition of cell migration and invasion of PC cells.[28]

Among the other differentially expressed miRNAs in responder patients, we observed miR-375-3p and miR-545-5p. Upregulation of miR-375-3p in PC cells resulted in inhibition of cell growth through PDK1 targeting and the regulation of Akt signaling pathway.[22] miR-545-5p decreases the PC cell growth through targeting of RIG-I, an intracellular viral RNA sensor involved in carcinogenesis.[29]

Figure 2. Characterization of surface extracellular vesicle (EV) markers

(A) Flow cytometry analysis of EV surface protein expression. Values refer to MFI median +/− 1–3 quartile of the most concentrated fractions. Blank control was used for normalization of the values. (B) Boxplots of EV protein expression analysis through flow cytometry in responder and non-responder patients. *p ≤ 0.05.

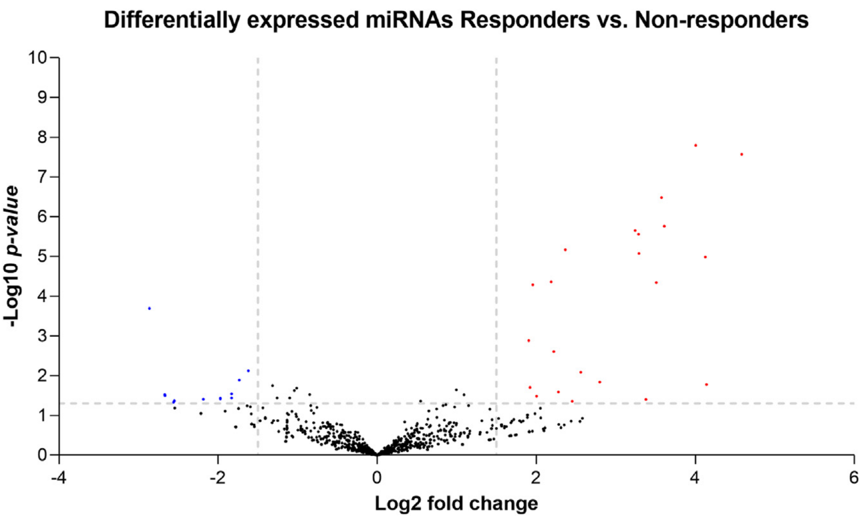

Figure 3. Volcano plot of differentially expressed miRNAs in EVs from responder and non-responders
The x axis represents the $\log_2$ fold change, and the y axis is the $-\log_{10}$ p value. The red colored dots represent miRNAs with a $\log_2$ fold change > 1.5 and $p < 0.05$. The blue colored dots show miRNAs with a $\log_2$ fold change < 1.5 and $p < 0.05$.

Other miRNAs were upregulated or downregulated in responder compared with non-responder patients; these miRNAs have not been described in PC literature and deserve to be explored in future research.

In conclusion, for the first time to our knowledge, we have found a different profile of the surface proteins and miRNA cargo of EVs isolated from advanced PC patients responding or not to first-line chemotherapy with gemcitabine + nab-paclitaxel, showing the feasibility of our approach for the identification of patients with more chances to benefit from therapy. This finding is worthy of further investigation, in a larger cohort of patients in the same setting, in patients treated with different chemotherapy schedules for advanced disease and in different PC settings such as preoperative treatment. Furthermore, our results indicate a deeper understanding of mechanisms of drug resistance, which includes analysis of both circulating factors and PC tissue, is required and will help therapy personalization strategies. Finally, our study may also serve for the development of EV-based delivery systems: indeed, the design of EVs to deliver specific miRNAs for new therapies represents a very promising strategy.

MATERIALS AND METHODS

Patients

The study population includes patients with metastatic or locally advanced pancreatic cancer, treated at IRCCS Istituto Romagnolo per lo Studio dei Tumori (IRST) "Dino Amadori," Meldola (FC), Italy. All patients had histologically or cytologically confirmed diagnosis of pancreatic adenocarcinoma, and received first-line treatment, in routine clinical practice, with gemcitabine + nab-paclitaxel: gemcitabine 1,000 mg/mg + nab-paclitaxel 125 mg/mq, d1,8,15 q28 (treatment schedule as in Von Hoff et al. [5]); if necessary, dose reductions were applied per standard clinical practice. All patients signed informed consent for collection of samples for translational research. Samples were collected before first-line treatment and stored in the local biobank facility. All baseline samples were collected between May 2015 and April 2021.

Tumor assessment was performed with thoracic-abdominal contrast-enhanced computed tomography scan, and tumor response was evaluated according to Response Evaluation Criteria in Solid Tumors (RECIST) 1.1.[31]

The present study has been approved by the local ethics committee (CEROM IRSTB118). The study complied with the provisions of the Good Clinical Practice guidelines and the Declaration of Helsinki and local laws and fulfilled Regulation (EU) 2016/679 of the European Parliament and the Council of April 27, 2016, on the protection of natural persons with regard to the processing of personal data.

Sample collection

Blood samples (5 mL) were collected in EDTA-containing tubes before starting treatment (in a time span of 14 days). Plasma was isolated within 2 h from blood withdrawal. Samples were centrifuged at 2000 x *g* for 15 min at room temperature in order to allow plasmatic fraction separation. Plasma was divided in cryogenic vials (0.5 mL each) and stored at −80°C until use.

EV isolation and RNA extraction

EVs were isolated from 1 mL of plasma by SEC columns of polysaccharide resin (qEV 70 columns; IZON, Christchurch, New Zealand) following the company's protocol. The EV-enriched fractions were collected. Subsequently, RNA was extracted from EVs by using the Plasma/Serum RNA Purification Mini Kit (Cat. 56100, Norgen Biotek, ON, Canada) as indicated in the manufacturer's protocol. The extracted RNAs were qualitatively evaluated with the Bioanalyzer 2100 instrument (Agilent Technologies, Milan, Italy) using RNA6000 pico chips.

NanoSight tracking analysis

The concentration (number/ml) and particle size (nm) of EVs was obtained through NTA. The analysis was performed with the instrument NanoSight NS300 (Malvern Instruments, Malvern, UK), equipped with NTA 2.3 analytical software laser. Prior to analysis, all samples were diluted in 0.1 μm filtered PBS, and subsequently three videos of 30 s each per sample were recorded at a camera level of 15 and in light scattering mode following the guidelines of the manufacturer. The NTA software version 2.3 was used to perform data analysis.

EV protein surface signature

Bead-based multiplex EV analysis by flow cytometry was used to characterize EV surface proteins (MACSPlex Exosome Kit, human;

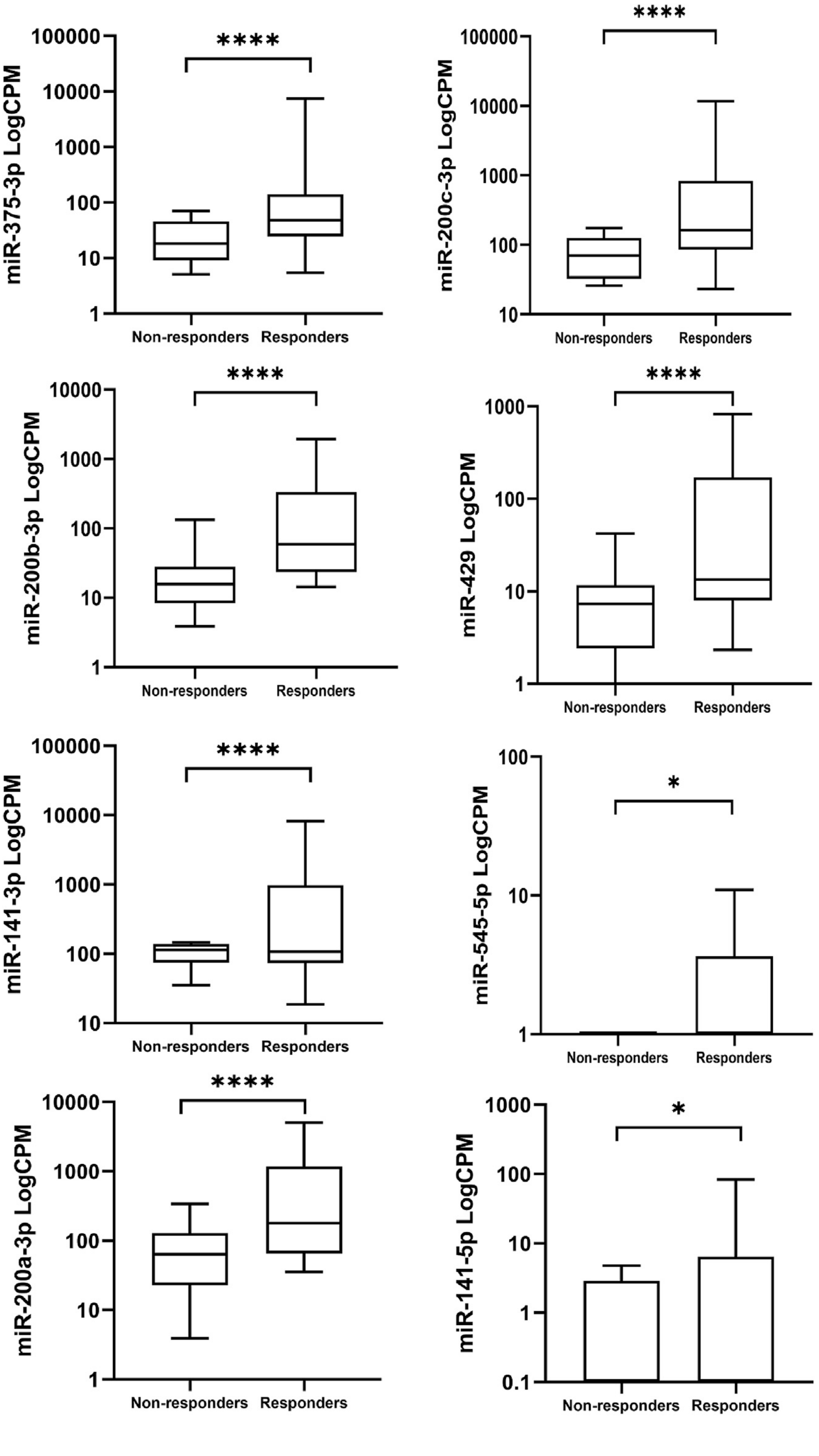

Figure 4. miRNA differential expression in responders and non-responders

Box plots of more differentially regulated miRNAs in responder and non-responder patients. The expression of each miRNA is indicated with logarithmic scale of counts per million. *p ≤ 0.05, ****p ≤ 0.0001.

MiltenyiBiotec, Bergisch Gladbach, Germany). This method allows us to analyze 37 different epitopes on the surface of the EVs including specific markers for the identification of exosomes (CD9, CD81, CD63). In brief, 70 μL of EV SEC eluate was diluted with MACSPlex buffer (MPB) to obtain a final volume of 120 μL. Each diluted sample was incubated for 1 h at room temperature on an orbital shaker at 450 rpm with different antibody-coated bead subsets and APC-conjugated anti-CD9, anti-CD63, and anti-CD81 detection antibodies. After some washes with MBP as described in the manufacturer's guidelines, the samples were analyzed with the cytometer (BD FACSCanto, BD Biosciences, Franklin Lakes, New Jersey, USA) obtaining the raw value of the MFI for each epitope. The MFI value of the negative control was subtracted from the obtained raw MFI value of each epitope.

microRNA profiling

Starting from 5 μL of total RNA, microRNA libraries were prepared using Qiaseq miRNA library kit (Qiagen, Düsseldorf, Germany). Libraries were prepared following manufacturer's instructions for low-input samples. Libraries were quantified using Qubit dsDNA HS assay kit (Thermo Fisher, Waltham, Massachusetts, USA) and quality checked on DNA high-sensitivity chips (Agilent Technologies, Santa Clara, California, USA). Normalized libraries were sequenced on NextSeq550 instrument (Illumina, San Diego, California, USA), approximately at a sequencing depth of 20 million reads per sample.

Bioinformatic and statistical analysis

Local RUN manager of NextSeq550 was used for demultiplexing. Reads were then trimmed, corrected for UMIs reduction, and aligned to mirBase v22 using the ready-to-use workflow for miRNA quantification of CLC Genomics Workbench, Biomedical Genomics analysis plugin (Qiagen, Düsseldorf, Germany). Data normalization (using the trimmed mean of M-values method) and differential expression analysis were performed using the CLC Genomic Workbench as well. Differentially expressed miRNAs were identified by setting the threshold $|\log2FC| > 1.5$ and $p < 0.05$ using multi-factorial statistics based on a negative binomial generalized linear model. Graphical representations were elaborated using GraphPad Prism 8 (Insight Partners, New York City, New York, USA).

Table 2. miRNA roles and targets

miRNA	Fold change	p value	Target	Function	Reference
miR-375-3p	4.58	2.685E-08	PDK1	tumor suppressor	Zhou et al.[22]
miR-200c-3p	4.01	1.593E-08	Zeb1	tumor suppressor	Yu et al. and Li et al.[23,24]
miR-200b-3p	3.24	2.220E-06	Zeb1	tumor suppressor	Li et al.; Wang et al. and Funamizu et al.[24–26]
miR-429	3.61	1.728E-06	TBK1	tumor suppressor	Song et al.[27]
miR-141-3p	3.58	3.298E-07	TM4SF1	tumor suppressor	Xu et al.[28]
miR-545-5p	3.38	3.949E-02	RIG-I	tumor suppressor	Song et al.[29]
miR-200a-3p	3.29	2.736E-06	β-catenin	tumor suppressor	Hu et al.[30]
miR-141-5p	2.80	1.443E-02	TM4SF1	tumor suppressor	Xu et al.[28]

Real-time PCR for miRNAs expression

To validate the miRNA sequencing results with real-time PCR, we evaluated the expression of two selected miRNAs: miR-200c-3p (ID 002300) and miR-375-3p (ID 000564). Since miR-16 emerged as equally expressed across samples in NGS data, hsa-miR-16 (ID 000391) was used as endogenous reference. In brief, 2 μL of total RNA extracted from EVs was converted into cDNA using the TaqMan microRNA RT kit (Thermo Fisher, Waltham, Massachusetts, USA), following the manufacturer's instructions. The real-time PCR reactions were run in triplicate on an ABI 7500 real-time PCR System (Thermo Fisher, Waltham, Massachusetts, USA) using TaqMan 2X Universal PCR Master Mix (Thermo Fisher, Waltham, Massachusetts, USA), following the thermal protocol suggested by the manufacturer. Relative expression of each target was calculated by normalizing the results to the endogenous control miR-16 with the $2^{-\Delta Ct}$ method. The Mann Whitney U test was used to compare the expression levels of miR-200c-3p and miR-375-3p between responder and non-responder PC patients. Graphical representations were elaborated using GraphPad Prism 8 (Insight Partners, New York City, New York, USA).

Statistical analysis

The comparison of the EV concentration, mean diameters, mode diameter, and the expression of the EV surface epitopes between patients was performed with the Mann Whitney U test. A two-sided testing was used to obtain all p values, which are considered significant with a value less than 0.05.

For survival analysis, overall survival (OS) was defined as the time interval from the first day of treatment to the day of death or last follow-up visit. Progression-free survival (PFS) was defined as the time interval from the first day of treatment to the day of tumor progression or death, whichever occurred first. OS and PFS were estimated by the Kaplan-Meier method.

SAS software, version 9.4 (SAS Institute, Cary, NC, USA) and R statistical package version 4.0.0 (R Foundation for Statistical Computing, Vienna, Austria) were used for data analysis.

DATA AVAILABILITY

The raw data have been deposited on the GEO public repository with the accession number GSE213341.

SUPPLEMENTAL INFORMATION

Supplemental information can be found online at https://doi.org/10.1016/j.omtm.2023.05.009.

ACKNOWLEDGMENTS

This work was partly supported thanks to the contribution of Ricerca Corrente by the Italian Ministry of Health within the research line "Precision, gender and ethnicity-based medicine and geroscience: genetic-molecular mechanisms in the development, characterization and treatment of tumors".

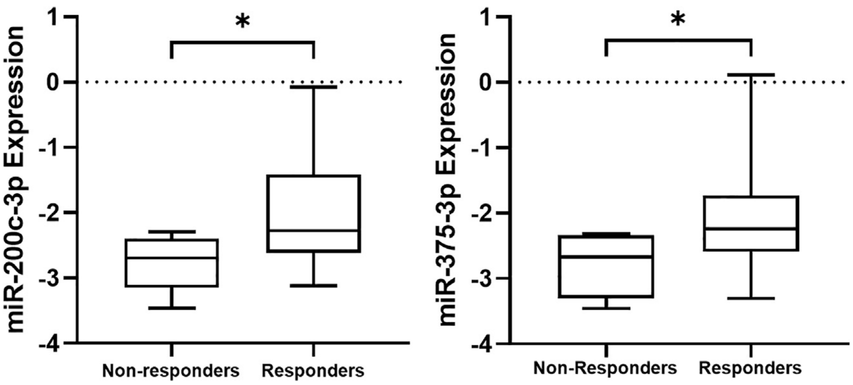

Figure 5. Expression of miR-200c-3p and miR-375-3p assessed by qPCR

Box plots depicting the expression ($\log(2^{-\Delta Ct})$) of miR-200c-3p (p = 0.0199; Mann-Whitney U test) and miR-375-3p (p = 0.046; Mann-Whitney U test) in responder and non-responder pancreatic cancer patients. The expression of miRNAs was evaluated by real-time PCR, and miR-16 was used as endogenous control. *p ≤ 0.05.

AUTHOR CONTRIBUTIONS

I.V. and I.G.R. contributed to the conceptualization and design of the study. I.V., T.R., M.M., and M.U. conducted the experiments. T.R. performed bioinformatic analysis. F.F. and S.B. performed cytometric analysis. I.A. performed statistical analysis. I.G.R., M.V., A.P., G.B., C.G., V.A., G.L.F., and L.M. contributed to data collection. I.V. and I.G.R. wrote the manuscript. All authors contributed to manuscript revision, read, and approved the submitted version.

DECLARATION OF INTERESTS

The authors declare that they have no competing interests.

REFERENCES

1. Global Burden of Disease Cancer Collaboration, Fitzmaurice, C., Abate, D., Abbasi, N., Abbastabar, H., Abd-Allah, F., Abdel-Rahman, O., Abdelalim, A., Abdoli, A., Abdollahpour, I., et al. (2019). Global, regional, and national cancer incidence, mortality, years of life lost, years lived with disability, and disability-adjusted life-years for 29 cancer groups, 1990 to 2017. JAMA Oncol. *5*, 1749–1768. https://doi.org/10.1001/jamaoncol.2019.2996.
2. Siegel, R.L., Miller, K.D., Fuchs, H.E., and Jemal, A. (2022). Cancer statistics, 2022. CA. Cancer J. Clin. *72*, 7–33. https://doi.org/10.3322/caac.21708.
3. Kindler, H.L. (2018). A glimmer of hope for pancreatic cancer. N. Engl. J. Med. *379*, 2463–2464. https://doi.org/10.1056/NEJMe1813684.
4. Conroy, T., Desseigne, F., Ychou, M., Bouché, O., Guimbaud, R., Bécouarn, Y., Adenis, A., Raoul, J.-L., Gourgou-Bourgade, S., de la Fouchardière, C., et al. (2011). FOLFIRINOX versus gemcitabine for metastatic pancreatic cancer. N. Engl. J. Med. *364*, 1817–1825. https://doi.org/10.1056/NEJMoa1011923.
5. Von Hoff, D.D., Ervin, T., Arena, F.P., Chiorean, E.G., Infante, J., Moore, M., Seay, T., Tjulandin, S.A., Ma, W.W., Saleh, M.N., et al. (2013). Increased survival in pancreatic cancer with nab-paclitaxel plus gemcitabine. N. Engl. J. Med. *369*, 1691–1703. https://doi.org/10.1056/NEJMoa1304369.
6. Reni, M., Zanon, S., Peretti, U., Chiaravalli, M., Barone, D., Pircher, C., Balzano, G., Macchini, M., Romi, S., Gritti, E., et al. (2018). Nab-paclitaxel plus gemcitabine with or without capecitabine and cisplatin in metastatic pancreatic adenocarcinoma (PACT-19): a randomised phase 2 trial. Lancet. Gastroenterol. Hepatol. *3*, 691–697. https://doi.org/10.1016/S2468-1253(18)30196-1.
7. Abrams, T.A., Meyer, G., Meyerhardt, J.A., Wolpin, B.M., Schrag, D., and Fuchs, C.S. (2017). Patterns of chemotherapy use in a U.S.-Based cohort of patients with metastatic pancreatic cancer. Oncologist *22*, 925–933. https://doi.org/10.1634/theoncologist.2016-0447.
8. Golan, T., Kanji, Z.S., Epelbaum, R., Devaud, N., Dagan, E., Holter, S., Aderka, D., Paluch-Shimon, S., Kaufman, B., Gershoni-Baruch, R., et al. (2014). Overall survival and clinical characteristics of pancreatic cancer in BRCA mutation carriers. Br. J. Cancer *111*, 1132–1138. https://doi.org/10.1038/bjc.2014.418.
9. Wattenberg, M.M., Asch, D., Yu, S., O'Dwyer, P.J., Domchek, S.M., Nathanson, K.L., Rosen, M.A., Beatty, G.L., Siegelman, E.S., and Reiss, K.A. (2020). Platinum response characteristics of patients with pancreatic ductal adenocarcinoma and a germline BRCA1, BRCA2 or PALB2 mutation. Br. J. Cancer *122*, 333–339. https://doi.org/10.1038/s41416-019-0582-7.
10. Golan, T., Hammel, P., Reni, M., Van Cutsem, E., Macarulla, T., Hall, M.J., Park, J.-O., Hochhauser, D., Arnold, D., Oh, D.-Y., et al. (2019). Maintenance olaparib for germline BRCA -mutated metastatic pancreatic cancer. N. Engl. J. Med. *381*, 317–327. https://doi.org/10.1056/NEJMoa1903387.
11. Heredia-Soto, V., Rodríguez-Salas, N., and Feliu, J. (2021). Liquid biopsy in pancreatic cancer: are we ready to apply it in the clinical practice? Cancers *13*, 1986. https://doi.org/10.3390/cancers13081986.
12. Becker, A., Thakur, B.K., Weiss, J.M., Kim, H.S., Peinado, H., and Lyden, D. (2016). Extracellular vesicles in cancer: cell-to-cell mediators of metastasis. Cancer Cell *30*, 836–848. https://doi.org/10.1016/j.ccell.2016.10.009.
13. Xu, R., Rai, A., Chen, M., Suwakulsiri, W., Greening, D.W., and Simpson, R.J. (2018). Extracellular vesicles in cancer — implications for future improvements in cancer care. Nat. Rev. Clin. Oncol. *15*, 617–638. https://doi.org/10.1038/s41571-018-0036-9.
14. Qiu, J., Yang, G., Feng, M., Zheng, S., Cao, Z., You, L., Zheng, L., Zhang, T., and Zhao, Y. (2018). Extracellular vesicles as mediators of the progression and chemoresistance of pancreatic cancer and their potential clinical applications. Mol. Cancer *17*, 2. https://doi.org/10.1186/s12943-017-0755-z.
15. Rawat, M., Kadian, K., Gupta, Y., Kumar, A., Chain, P.S.G., Kovbasnjuk, O., Kumar, S., and Parasher, G. (2019). MicroRNA in pancreatic cancer: from biology to therapeutic potential. Genes *10*, 752. https://doi.org/10.3390/genes10100752.
16. Melo, S.A., Sugimoto, H., O'Connell, J.T., Kato, N., Villanueva, A., Vidal, A., Qiu, L., Vitkin, E., Perelman, L.T., Melo, C.A., et al. (2014). Cancer exosomes perform cell-independent MicroRNA biogenesis and promote tumorigenesis. Cancer Cell *26*, 707–721. https://doi.org/10.1016/j.ccell.2014.09.005.
17. Si, W., Shen, J., Zheng, H., and Fan, W. (2019). The role and mechanisms of action of microRNAs in cancer drug resistance. Clin. Epigenet. *11*, 25. https://doi.org/10.1186/s13148-018-0587-8.
18. Patel, G.K., Khan, M.A., Bhardwaj, A., Srivastava, S.K., Zubair, H., Patton, M.C., Singh, S., Khushman, M., and Singh, A.P. (2017). Exosomes confer chemoresistance to pancreatic cancer cells by promoting ROS detoxification and miR-155-mediated suppression of key gemcitabine-metabolising enzyme, DCK. Br. J. Cancer *116*, 609–619. https://doi.org/10.1038/bjc.2017.18.
19. Richards, K.E., Zeleniak, A.E., Fishel, M.L., Wu, J., Littlepage, L.E., and Hill, R. (2017). Cancer-associated fibroblast exosomes regulate survival and proliferation of pancreatic cancer cells. Oncogene *36*, 1770–1778. https://doi.org/10.1038/onc.2016.353.
20. Pasini, L., Vannini, I., Ulivi, P., Tebaldi, M., Petracci, E., Fabbri, F., Stella, F., and Urbini, M. (2022). Comparative analysis of free-circulating and vesicle-associated plasma microRNAs of healthy controls and early-stage lung cancer patients. Pharmaceutics *14*, 2029. https://doi.org/10.3390/pharmaceutics14102029.
21. Wiklander, O.P.B., Bostancioglu, R.B., Welsh, J.A., Zickler, A.M., Murke, F., Corso, G., Felldin, U., Hagey, D.W., Evertsson, B., Liang, X.-M., et al. (2018). Systematic methodological evaluation of a multiplex bead-based flow cytometry assay for detection of extracellular vesicle surface signatures. Front. Immunol. *9*, 1326. https://doi.org/10.3389/fimmu.2018.01326.
22. Zhou, J., Song, S., He, S., Zhu, X., Zhang, Y., Yi, B., Zhang, B., Qin, G., and Li, D. (2014). MicroRNA-375 targets PDK1 in pancreatic carcinoma and suppresses cell growth through the Akt signaling pathway. Int. J. Mol. Med. *33*, 950–956. https://doi.org/10.3892/ijmm.2014.1638.
23. Yu, J., Ohuchida, K., Mizumoto, K., Sato, N., Kayashima, T., Fujita, H., Nakata, K., and Tanaka, M. (2010). MicroRNA, hsa-miR-200c, is an independent prognostic factor in pancreatic cancer and its upregulation inhibits pancreatic cancer invasion but increases cell proliferation. Mol. Cancer *9*, 169. https://doi.org/10.1186/1476-4598-9-169.
24. Li, Y., VandenBoom, T.G., Kong, D., Wang, Z., Ali, S., Philip, P.A., and Sarkar, F.H. (2009). Up-regulation of miR-200 and let-7 by natural agents leads to the reversal of epithelial-to-mesenchymal transition in gemcitabine-resistant pancreatic cancer cells. Cancer Res. *69*, 6704–6712. https://doi.org/10.1158/0008-5472.CAN-09-1298.
25. Wang, Z., Chen, Y., Lin, Y., Wang, X., Cui, X., Zhang, Z., Xian, G., and Qin, C. (2017). Novel crosstalk between KLF4 and ZEB1 regulates gemcitabine resistance in pancreatic ductal adenocarcinoma. Int. J. Oncol. *51*, 1239–1248. https://doi.org/10.3892/ijo.2017.4099.
26. Funamizu, N., Lacy, C.R., Kamada, M., Yanaga, K., and Manome, Y. (2019). MicroRNA-200b and -301 are associated with gemcitabine response as biomarkers in pancreatic carcinoma cells. Int. J. Oncol. *54*, 991–1000. https://doi.org/10.3892/ijo.2019.4676.
27. Song, B., Zheng, K., Ma, H., Liu, A., Jing, W., Shao, C., Li, G., and Jin, G. (2015). MiR-429 determines poor outcome and inhibits pancreatic ductal adenocarcinoma growth by targeting TBK1. Cell. Physiol. Biochem. *35*, 1846–1856. https://doi.org/10.1159/000373995.
28. Xu, L., Li, Q., Xu, D., Wang, Q., An, Y., Du, Q., Zhang, J., Zhu, Y., and Miao, Y. (2014). hsa-miR-141 downregulates TM4SF1 to inhibit pancreatic cancer cell invasion and migration. Int. J. Oncol. *44*, 459–466. https://doi.org/10.3892/ijo.2013.2189.

29. Song, B., Ji, W., Guo, S., Liu, A., Jing, W., Shao, C., Li, G., and Jin, G. (2014). miR-545 inhibited pancreatic ductal adenocarcinoma growth by targeting RIG-I. FEBS Lett. *588*, 4375–4381. https://doi.org/10.1016/j.febslet.2014.10.004.

30. Hu, B., Qiu-lan, H., Lei, R.E., Shi, C., Jiang, H.X., and Qin, S.Y. (2017). Interleukin-9 promotes pancreatic cancer cells proliferation and migration via the miR-200a/beta-catenin Axis. BioMed Res. Int. *2017*, 2831056–2831059. https://doi.org/10.1155/2017/2831056.

31. Eisenhauer, E.A., Therasse, P., Bogaerts, J., Schwartz, L.H., Sargent, D., Ford, R., Dancey, J., Arbuck, S., Gwyther, S., Mooney, M., et al. (2009). New response evaluation criteria in solid tumours: revised RECIST guideline (version 1.1). Eur. J. Cancer *45*, 228–247. https://doi.org/10.1016/j.ejca.2008.10.026.

32. Kannagi, R., Cochran, N.A., Ishigami, F., Hakomori, S., Andrews, P.W., Knowles, B.B., and Solter, D. (1983). Stage-specific embryonic antigens (SSEA-3 and -4) are epitopes of a unique globo-series ganglioside isolated from human teratocarcinoma cells. EMBO J. *2*, 2355–2361. https://doi.org/10.1002/j.1460-2075.1983.tb01746.x.

33. Shamblott, M.J., Axelman, J., Wang, S., Bugg, E.M., Littlefield, J.W., Donovan, P.J., Blumenthal, P.D., Huggins, G.R., and Gearhart, J.D. (1998). Derivation of pluripotent stem cells from cultured human primordial germ cells. Proc. Natl. Acad. Sci. USA *95*, 13726–13731. https://doi.org/10.1073/pnas.95.23.13726.

34. Lin, C.-W., Wang, Y.-J., Lai, T.-Y., Hsu, T.-L., Han, S.-Y., Wu, H.-C., Shen, C.-N., Dang, V., Chen, M.-W., Chen, L.-B., and Wong, C.H. (2021). Homogeneous antibody and CAR-T cells with improved effector functions targeting SSEA-4 glycan on pancreatic cancer. Proc. Natl. Acad. Sci. USA *118*. e2114774118. https://doi.org/10.1073/pnas.2114774118.

35. Quagliano, A., Gopalakrishnapillai, A., Kolb, E.A., and Barwe, S.P. (2020). CD81 knockout promotes chemosensitivity and disrupts in vivo homing and engraftment in acute lymphoblastic leukemia. Blood Adv. *4*, 4393–4405. https://doi.org/10.1182/bloodadvances.2020001592.

36. Klicka, K., Grzywa, T.M., Mielniczuk, A., Klinke, A., and Włodarski, P.K. (2022). The role of miR-200 family in the regulation of hallmarks of cancer. Front. Oncol. *12*, 965231. https://doi.org/10.3389/fonc.2022.965231.

OMTM, Volume *29*

Supplemental information

Analysis of EVs from patients with advanced pancreatic cancer identifies antigens and miRNAs with predictive value

Ivan Vannini, Tania Rossi, Mattia Melloni, Martina Valgiusti, Milena Urbini, Alessandro Passardi, Giulia Bartolini, Chiara Gallio, Irene Azzali, Sara Bandini, Valentina Ancarani, Lorenzo Montanaro, Giovanni Luca Frassineti, Francesco Fabbri, and Ilario Giovanni Rapposelli

Supplemental Material

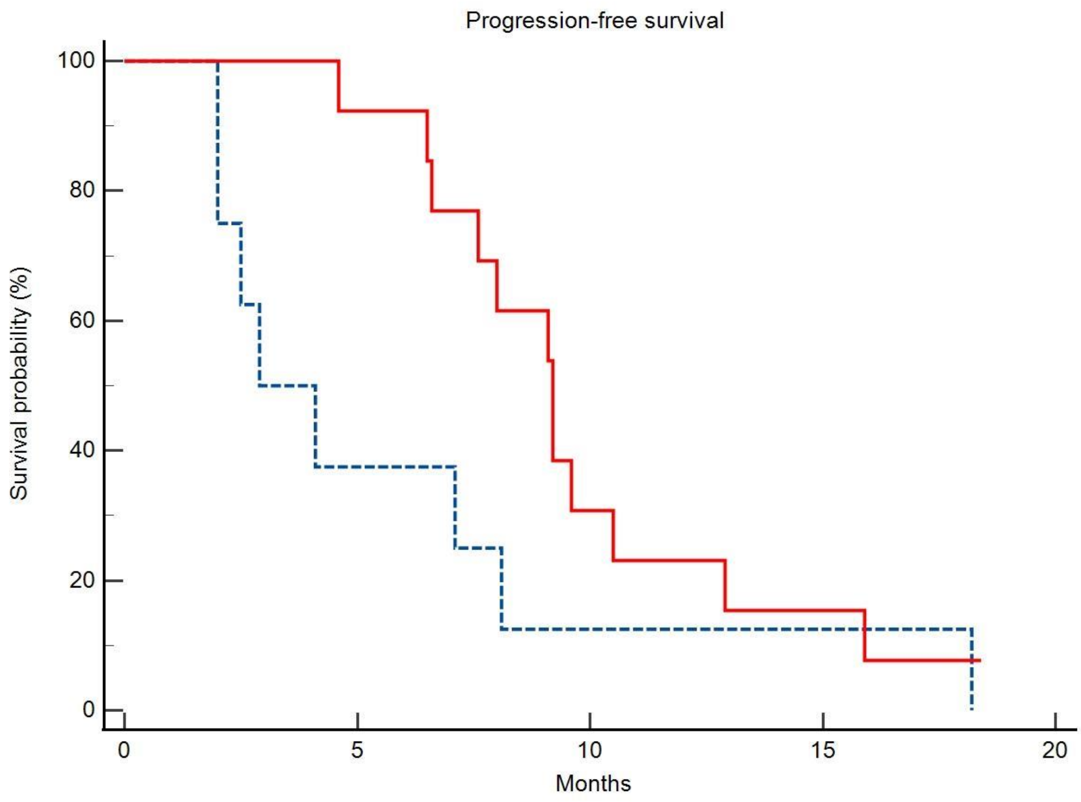

Figure S1. Kaplan-Meier analysis of progression-free survival. Median progression-free survival was 9.2 months for responders and 2.9 months for non-responders (hazard ratio 0.39, 95% confidence interval 0.13-1.12; p = 0.08). Red line: responders; blue line: non-responders.

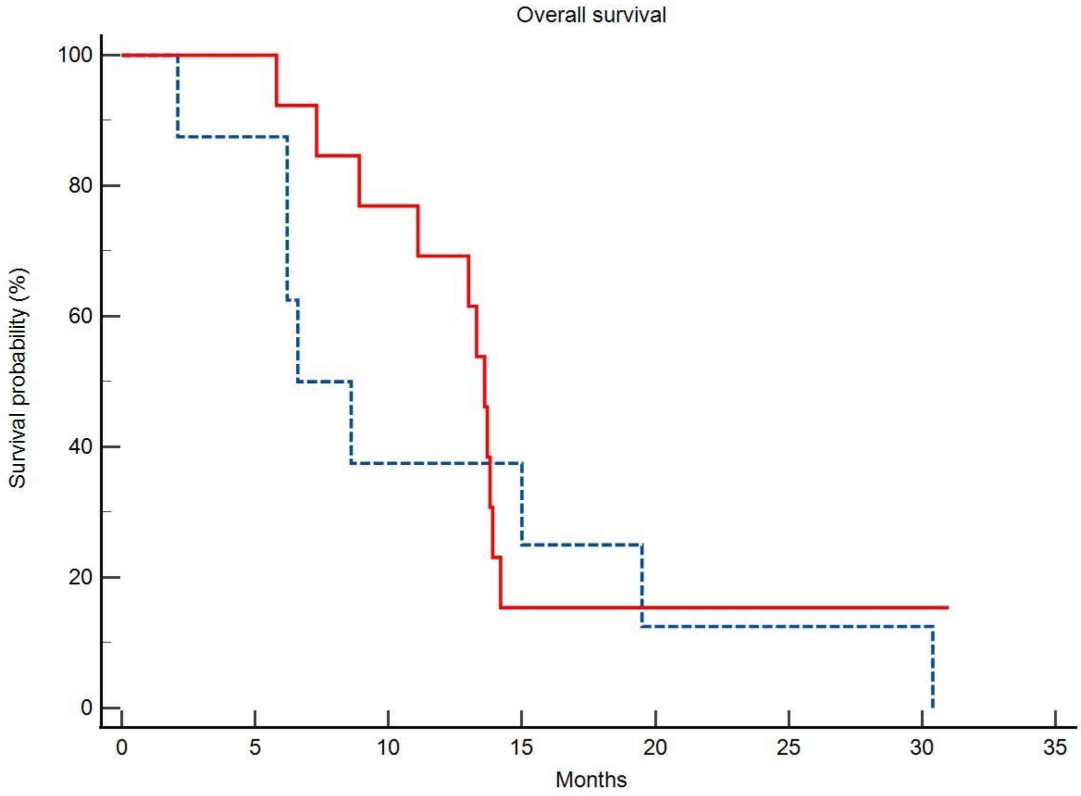

Figure S2. Kaplan-Meier analysis of overall survival. Median overall survival was 13.6 months for responders and 6.6 months for non-responders (hazard ratio 0.77, 95% confidence interval 0.29-2.04; p = 0.60). Red line: responders; blue line: non-responders.

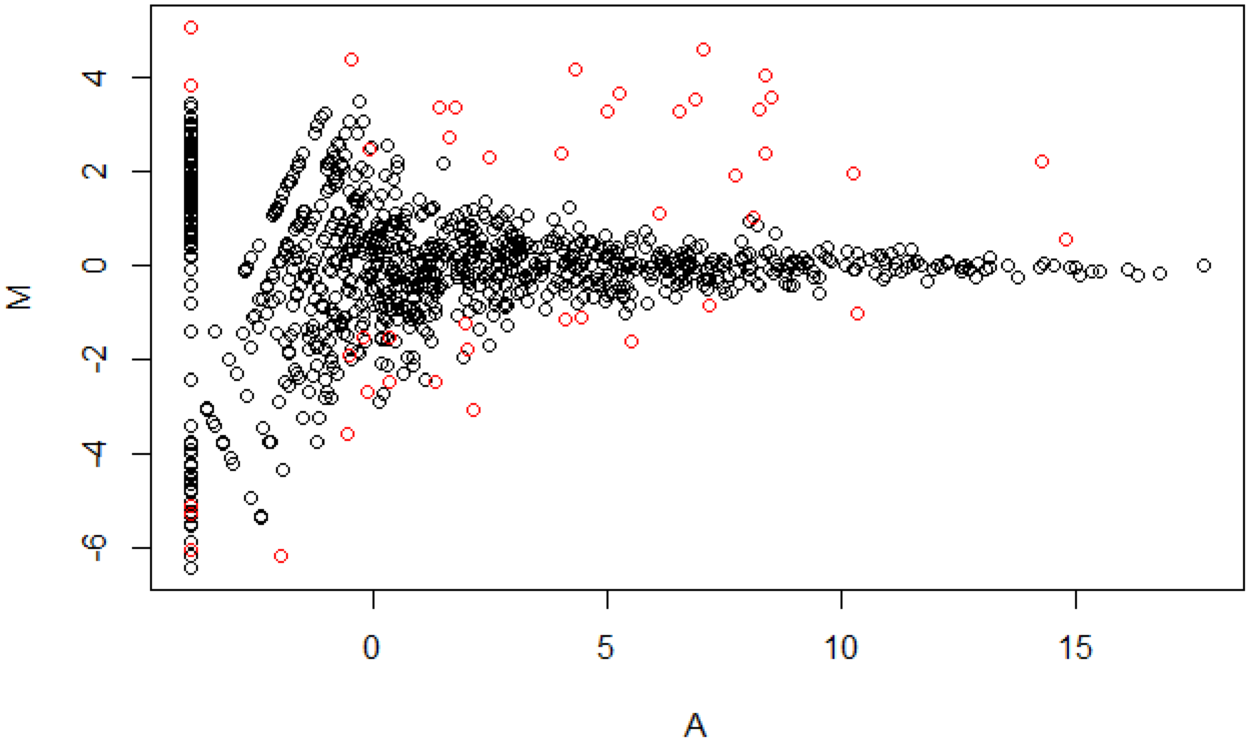

Figure S3. MA-plot displaying the log fold change (M) against the log abundance (A) of microRNAs (miRNAs). Red dots identify the differentially expressed miRNAs (p-value < 0.05) between responder and non-responder advanced pancreatic cancer patients, as described in the main text.

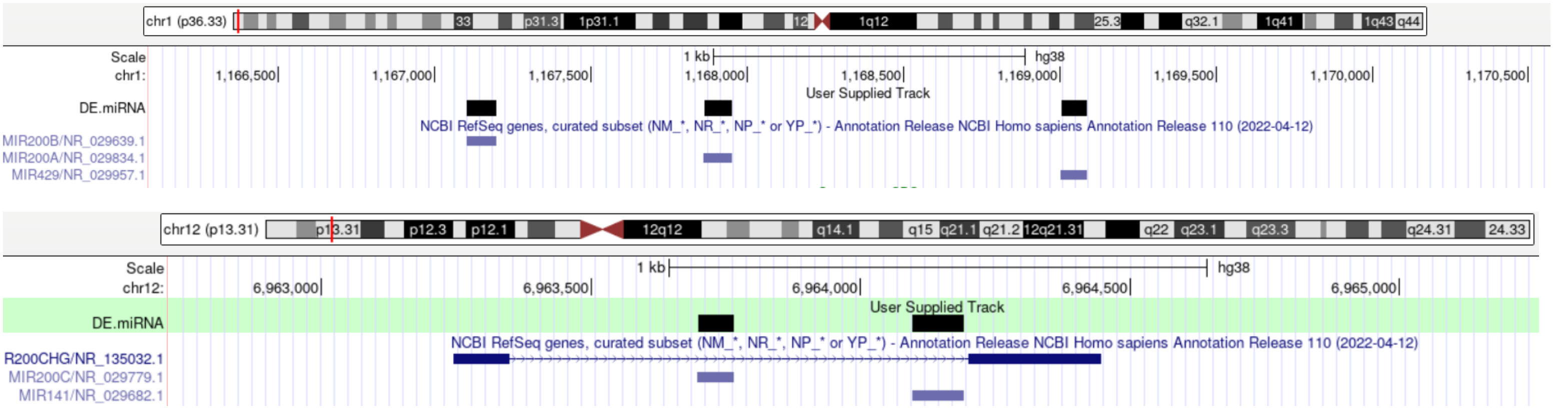

Figure S4. Genomic localization of miR-200 family members in chromosome 1 (miR-200a, miR-200b, miR-429) and chromosome 12 (miR-200c, miR-141). "DE.miRNA"= UCSC tracks of differentially expressed miRNAs found in responder vs non-responser PC patients.

Table S1. List of differentially expressed microRNAs (miRNAs) between responder and non-responder pancreatic cancer patients.

miRNA	Fold change	Log fold change	*P*-value	Genomic position (GRCh38) Chromosome: start-end [strand]
hsa-miR-375-3p	23,98	4,58	2,685E-08	chr2: 219001645-219001708 [-]
hsa-miR-200a-5p	17,63	4,14	1,682E-02	chr1: 1167863-1167952 [+]
hsa-miR-135b-5p	17,47	4,13	1,029E-05	chr1: 205448302-205448398 [-]
hsa-miR-200c-3p	16,07	4,01	1,593E-08	chr12: 6963699-6963766 [+]
hsa-miR-429	12,22	3,61	1,728E-06	chr1: 1169005-1169087 [+]
hsa-miR-141-3p	11,92	3,58	3,298E-07	chr12: 6964097-6964191 [+]
hsa-miR-196a-5p	11,40	3,51	4,546E-05	chr17: 48632490-48632559 [-]
hsa-miR-545-5p	10,40	3,38	3,949E-02	chrX: 74287104-74287209 [-]
hsa-miR-215-5p	9,79	3,29	8,493E-06	chr1: 220117853-220117962 [-]
hsa-miR-200a-3p	9,76	3,29	2,736E-06	chr1: 1167863-1167952 [+]
hsa-miR-200b-3p	9,46	3,24	2,220E-06	chr1: 1167104-1167198 [+]
hsa-miR-141-5p	6,96	2,80	1,443E-02	chr12: 6964097-6964191 [+]
hsa-miR-885-3p	5,90	2,56	8,151E-03	chr3: 10394489-10394562 [-]
hsa-miR-1268a	5,47	2,45	4,396E-02	chr15: 22225278-22225329 [-]
hsa-miR-194-5p	5,15	2,37	6,794E-06	chr1: 220118157-220118241 [-]
hsa-miR-200b-5p	4,85	2,28	2,589E-02	chr1: 1167104-1167198 [+]
hsa-miR-122-3p	4,66	2,22	2,479E-03	chr18: 58451074-58451158 [+]
hsa-miR-122-5p	4,56	2,19	4,346E-05	chr18: 58451074-58451158 [+]
hsa-miR-589-3p	4,01	2,00	3,304E-02	chr7: 5495819-5495917 [-]
hsa-miR-192-5p	3,88	1,96	5,158E-05	chr11: 64891137-64891246 [-]
hsa-miR-455-5p	3,79	1,92	1,972E-02	chr9: 114209434-114209529 [+]
hsa-miR-92b-3p	3,75	1,91	1,306E-03	chr1: 155195177-155195272 [+]
hsa-miR-100-5p	2,13	1,09	3,032E-02	chr11: 122152229-122152308 [-]
hsa-miR-483-5p	2,00	1,00	2,277E-02	chr11: 2134134-2134209 [-]
hsa-miR-21-5p	1,46	0,55	4,339E-02	chr17: 59841266-59841337 [+]
hsa-miR-411-5p	-1,80	-0,85	2,981E-02	chr14: 101023325-101023420 [+]
hsa-miR-125b-5p	-2,02	-1,01	2,062E-02	chr11: 122099757-122099844 [-]
hsa-miR-136-5p	-2,06	-1,04	2,383E-02	chr14: 100884702-100884783 [+]
hsa-miR-1249-3p	-2,14	-1,10	3,642E-02	chr22: 45200954-45201019 [-]
hsa-miR-19b-1-5p	-2,39	-1,26	3,636E-02	chr13: 91351192-91351278 [+]
hsa-miR-1185-1-3p	-2,49	-1,32	1,769E-02	chr14: 101042977-101043062 [+]
hsa-miR-190b-5p	-3,07	-1,62	7,568E-03	chr1: 154193665-154193743 [-]
hsa-miR-496	-3,32	-1,73	1,291E-02	chr14: 101060573-101060674 [+]
hsa-miR-18b-5p	-3,56	-1,83	3,640E-02	chrX: 134170041-134170111 [-]
hsa-miR-12135	-3,56	-1,83	2,872E-02	chr15: 73299910-73299979 [+]
hsa-miR-6131	-3,93	-1,97	3,826E-02	chr5: 10478037-10478145 [+]
hsa-miR-516a-5p	-3,93	-1,97	3,690E-02	chr19: 53756741-53756830 [+]
hsa-miR-3161	-4,55	-2,19	3,920E-02	chr11: 48096782-48096858 [+]
hsa-miR-6806-3p	-5,86	-2,55	4,309E-02	chr19: 58334688-58334751 [+]
hsa-miR-4451	-5,86	-2,55	4,307E-02	chr4: 85722468-85722533 [+]
hsa-miR-411-3p	-5,90	-2,56	4,681E-02	chr14: 101023325-101023420 [+]
hsa-miR-216a-5p	-6,36	-2,67	3,178E-02	chr2: 55988950-55989059 [-]
hsa-miR-362-3p	-6,37	-2,67	2,990E-02	chrX: 50008964-50009028 [+]
hsa-miR-146a-3p	-7,28	-2,86	2,022E-04	chr5: 160485352-160485450 [+]
